# Comparison of Scalp ERP to Faces in Macaques and Humans

**DOI:** 10.3389/fnsys.2021.667611

**Published:** 2021-04-16

**Authors:** John Orczyk, Charles E. Schroeder, Ilana Y. Abeles, Manuel Gomez-Ramirez, Pamela D. Butler, Yoshinao Kajikawa

**Affiliations:** ^1^Translational Neuroscience Division, Center for Biological Imaging and Neuromodulation, Nathan Kline Institute for Psychiatric Research, Orangeburg, NY, United States; ^2^Department of Neurological Surgery, Vagelos College of Physicians and Surgeons, Columbia University Medical Center, New York, NY, United States; ^3^Clinical Research Department, Nathan Kline Institute for Psychiatric Research, Orangeburg, NY, United States; ^4^Psychiatry Department, School of Medicine, New York University, New York, NY, United States

**Keywords:** face response, ERP, N170, macaque monkey, superior temporal sulcus, fusiform face area

## Abstract

Face recognition is an essential activity of social living, common to many primate species. Underlying processes in the brain have been investigated using various techniques and compared between species. Functional imaging studies have shown face-selective cortical regions and their degree of correspondence across species. However, the temporal dynamics of face processing, particularly processing speed, are likely different between them. Across sensory modalities activation of primary sensory cortices in macaque monkeys occurs at about 3/5 the latency of corresponding activation in humans, though this human simian difference may diminish or disappear in higher cortical regions. We recorded scalp event-related potentials (ERPs) to presentation of *faces* in macaques and estimated the peak latency of ERP components. Comparisons of latencies between macaques (112 ms) and humans (192 ms) suggested that the 3:5 ratio could be preserved in higher cognitive regions of face processing between those species.

## Introduction

Social mammals perceive and recognize faces swiftly and automatically, an ability crucial for social living (Leopold and Rhodes, 2010). While early studies identified face-responsive neurons in the superior temporal regions of monkeys (Gross et al., 1972; Perrett et al., 1982; Desimone et al., 1984), brain mechanisms of face perception are investigated under various behavioral contexts using non-invasive techniques in both monkeys and humans (Kanwisher and Yovel, 2006; Rossion, 2014). Functional MRI studies consistently found face-selective areas in the typical regions, the fusiform face area (FFA), occipital face area (OFA) and posterior superior temporal sulcus (STS; Kanwisher and Yovel, 2006; Tsao and Livingstone, 2008; Yovel and Freiwald, 2013). The onset of face stimuli evokes robust responses in the scalp event-related potential (ERP) with temporal peak components around 170 ms, with topographic distributions composed of positive peaks, the vertex positive potential or VPP at the central midline, and the negative peak N170 at the occipitotemporal surface (Botzel and Grusser, 1989; Jeffreys, 1989; Bentin et al., 1996; Joyce and Rossion, 2005). Sources of VPP and N170 were generally found in FFA or OFA as well (Rossion et al., 2003; Kanwisher and Yovel, 2006).

Behavioral testing suggests non-human primates perceive faces in a manner similar to humans (Pascalis and Bachevalier, 1998; Leopold and Rhodes, 2010; Kazem and Widdig, 2013; Taubert et al., 2017). While information processed for face perception may differ between species (Martin-Malivel et al., 2006; Parr et al., 2009; Rossion and Taubert, 2009), electrophysiological studies found face responses over wide ranges of the superior temporal sulcus (STS) in a number of behavioral conditions (Desimone et al., 1984; Tanaka et al., 1991; Sugase et al., 1999; Tsao et al., 2003; Kajikawa et al., 2017). These findings support the idea that STS activation constitutes a large source of electrical activity. Macaque brain imaging studies found face-selective patches in cortices along STS and inferior temporal gyrus, a fraction of those were suggested to be homologous to human FFA or OFA (Tsao et al., 2008). Selective unit responses to faces have been found consistently in these cortices (Perrett et al., 1982; Desimone et al., 1984; Tsao et al., 2006; Freiwald and Tsao, 2010). While both primate species have been studied extensively for their face responses, the main electrophysiological techniques used in human studies, measuring ERPs, differ significantly in scope and resolution from the single unit recordings used in most macaque studies. Only one study has examined scalp ERPs to faces in macaque monkeys (Pineda and Nava, 1993). While this study examined the effects of different face presentation conditions, they did not address the neural sources of ERP components. Based on findings of previous studies, we considered STS as a main source of face responses, and placed scalp electrodes to focus on detecting STS activity, accordingly.

In the present study, we recorded scalp ERPs from two monkeys performing audiovisual tasks, in which a fraction of trials required animals to monitor repeatedly presented monkey face stimuli without sound, requiring only visual attention, and discriminate oddball faces (Kajikawa et al., 2017). We recorded ERPs using a pair of electrodes that flank the STS and have the same orientation as cortical columns of STS to enhance detection of ERP generated by cortices in the banks of STS. We compared monkey scalp ERPs with human scalp ERPs to human face stimuli presented in an equivalent experimental condition. Our results show that monkeys’ face-ERPs have P1-N1-P2 components similar to humans’ face-ERPs. They also show that a customary adjustment for macaque-human latency difference, the “3/5ths rule,” applies to the more complex face-evoked responses generated in higher order brain regions, just as it does to simpler stimulus evoked responses generated in primary sensory areas (Schroeder et al., 1995, 2004).

## Materials and Methods

### Monkey Subjects

All procedures were approved by the Institutional Animal Care and Use Committee of the Nathan Kline Institute (NKI) and conducted in compliance with the Guide for the Care and Use of Laboratory Animals (National Research Council of the National Academies). Two male macaque monkeys (*Macaca mulatta*; G, 11 kg; W, 9 kg) were implanted with headposts using aseptic surgical techniques. These monkeys also served in our previous studies of intracortical responses to faces (Kajikawa et al., 2017). Scalp ERP recordings in the present study were made before implantation of chambers for the subsequent intracortical recordings.

### Stimuli (Monkey Experiments)

Eight exemplars of macaque vocalization movie clips (29.97 fps for visual, 44.1 kHz for auditory) were used. The clips were edited to show monkey faces occupying an area of 10 degrees in diameter. The clip started with the onset of vocalizing face movements and lasted for 500 ms, using Adobe Premiere (Adobe Systems, San Jose, CA, USA), and separated into video and audio tracks using the utility software of Experiment Builder (SR-Research). The background of the faces in visual tracks was blackened.

For the static images that appeared at the beginning of trials and between vocalization clips during trials, the first frames of edited visual tracks were also saved separately as an image. Scrambles of non-black pixels in the first frame images were constructed using Matlab. Either the scrambled images (*Task 1*) or intact first frame images (*Task 2*) were used as static images that appeared before movie clips. In AV and V-alone trials, Movie clips were presented with (AV trials) or without timing-matched vocal sounds (V-alone trials).

All visual stimuli were presented on a monitor (FlexScan F930, EIZO), 90 cm in front of monkeys. Images and movies were presented in the rectangle window (17.8 × 11.4 degrees) at the center of a blank screen. Auditory stimuli (vocal sounds for *Tasks 1* and *2*, and pure tones for *Task 3*) were delivered from either loudspeakers (Tannoy Precision 6P) placed on both sides of the monitor through an amplifier (Ashly ne800) for monkey W or through magnetic speakers (FF1, Tucker-Davies Technologies, Alachua, FL, USA) placed at 4 inches from ears.

### Behavioral Procedures (Monkey Experiments)

**Figure 1:**
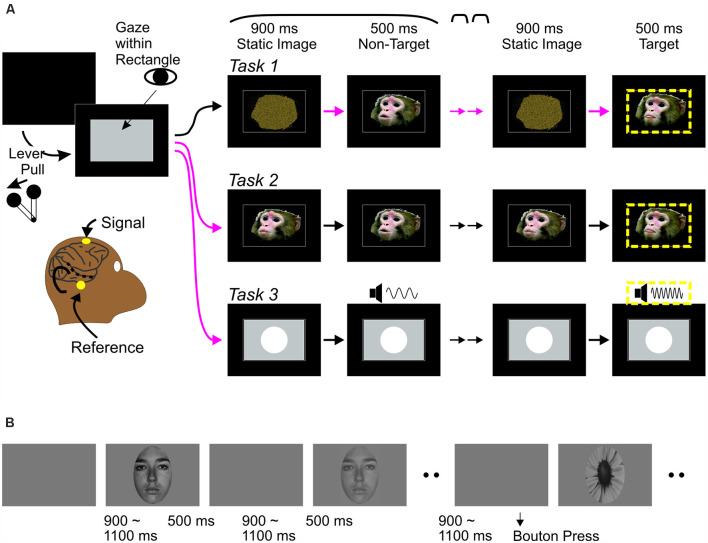
Behavioral paradigms. **(A)** Monkey experiments: event sequences during visual-alone trials of audiovisual tasks (*Task 1* and *2*) and trials of *Task 3*. In all tasks, a trial was initiated when the monkey pulled a lever, bringing up a rectangular gray window on a monitor screen, followed by a repetition of a static image—non-target stimulus (a movie clip on screen for *Tasks 1* and *2*, and a pure tone while the screen remains static for *Task 3*) sequence. After repeating the sequence randomly from three to six times, an oddball stimulus occurred, this was a target (circumscribed by yellow dashed lines). Monkeys had to release the lever upon detection of the target to receive a reward. *Tasks 1–3* differed in the static image at the beginning of trials. In *Task 1*, all trials started with a scrambled image. In *Task 2*, all trials started with a static face image. Magenta arrows indicate the timing when abrupt onset of faces occurred in each trial. The face onsets may occur three to six times in *Task 1*, but only once in *Task 2*. In *Task 3*, a circle appeared occurred abruptly once in each trial like the face onset in *Task 2*. While all abrupt changes related to stimulus in screen were potential behavioral targets in *Task 1*, an abrupt change on screen occurring once early in each trial was not in *Tasks 2* and *3*. **(B)** Human experiments: Face (3 contrast levels) and target stimuli were presented randomly, with 9:1 ratio of frequencies. Participants were instructed to press a bouton at the onset of the target (flower) and ignore all other stimuli.

All tasks were variations of a classic “oddball” task widely used in human studies, in which a single stimulus is repeated several times (standard stimulus) in a stream, inserted with occasional deviant (“oddball” stimulus). The oddballs served as targets for behavioral responses, ensuring that animals pay attention to all stimuli. There were two versions of the audiovisual task (*Tasks 1 and 2*) that differed in static images before and between movie clips (see *Stimuli*). Otherwise, both tasks proceeded in the same manner (Figure 1). The monkey pulled a lever on the monkey chair to bring up a gray rectangular window on the screen. The monkey then maintained gaze position within the window for at least 400 ms to initiate a trial. Gaze had to be maintained through a target stimulus. A trial started with a static image appearing in the window for 900 ms, followed by 3–6 presentations of a 500 ms vocalization clip (“standard,” non-target stimuli) and an oddball clip that served as the target in each trial. The same static image appeared between clips for durations randomly between values of 600, 750, 900, 1,050 and 1,200 ms. Clips were presented in three conditions: a-alone, V-alone, or AV (see Kajikawa et al., 2017 for detail). Oddball clips differed from the standards in one (A or V) or both modalities. The monkeys responded to target detection manually by releasing the lever to obtain a liquid reward. Each manual response was followed by *a* > 1 s blank period. Gaze position was monitored using Eyelink-1000 (SR-Research). Stimulus deliveries, tracking of the lever, and reward deliveries were controlled using Experiment Builder (SR-Research). In auditory oddball tasks (*Task 3*), screen images and movies were replaced by static image of a circle (8.3 degrees) through the period of trials, while 300 ms pure tones (60 dB) were repeatedly delivered with 600–1,200 ms intervals, followed by an oddball tone of different pitch.

### Electrophysiological Recordings (Monkey Experiments)

**Figure 2:**
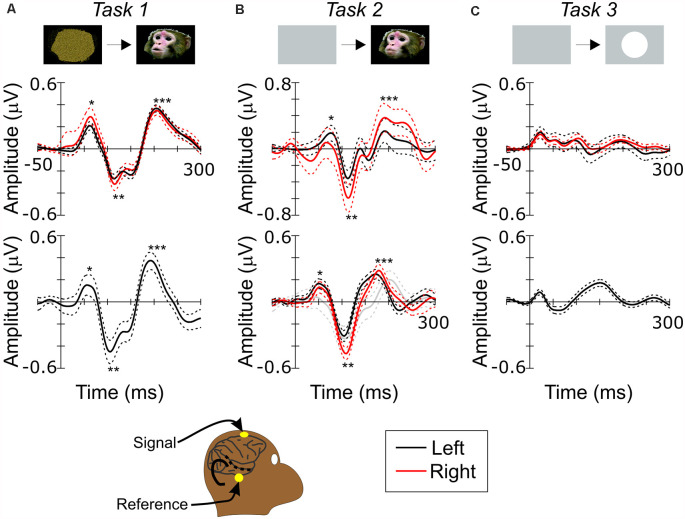
Scalp recording of visual responses in monkeys. **(A)** Scalp ERP to face during *Task 1*. **(B)** Scalp ERP to face during *Task 2*. **(C)** Scalp ERP to circle during *Task 3*. Top and bottom rows show responses in monkey G and W, respectively. Black and red lines show responses recorded in left and right hemispheres, respectively. Dashed lines are 95% confidence intervals. Single, double and triple asterisks in **(A)** and **(B)** label P1, N1 and P2 components. Bottom inset depicts a schematic diagram of scalp recording positions relative to Superior Temporal Sulcus (STS) on right side of monkey’s head. Gray contour outlines brain and sulci. Black dashed line depicts STS. Superimposed yellow dots indicate the electrode positions.

All recordings were conducted while the monkeys performed the tasks. Two gold plated EEG electrodes (Grass Instruments) filled with conductive gel were placed on scalp, one at the FCz and the other above zygomatic arch in front of an ear, as signal and reference electrodes, respectively (Figure 2A). Those positions are above and below STS (Kajikawa et al., 2015). The differential electrical potential was amplified 2,000× for Monkey G and 5,000× for Monkey W (BMA-400, CWE Inc.), and digitized at 2,000 Hz. Line noise was filtered offline using a fir notch filter implemented in Matlab, as needed.

### Data Analysis (Monkey Experiments)

Responses to stimuli were calculated by averaging EEG signals across all stimulus presentation trials. Signals were digitally bandpass filtered offline using a third order Butterworth filter at cutoff frequencies of 1 and 256 Hz. Peak amplitudes were estimated after subtracting the mean amplitude of a 50 ms baseline period. Confidence intervals of waveforms were derived using bootstrap (1,000 resamples) of 3,251, 1,379 and 4,662 trials in Monkey G and 1,860, 1,912 and 1,929 trials in Monkey W, respectively for *Tasks 1*, *2*, and *3*. To compare peak amplitudes, bootstrap test of difference in the mean between two samples was used (Efron and Tibshirani, 1993).

### Human Subjects

Twenty-one healthy volunteers (15 males/6 females; Mean age 38.4 ± 12.8 years) with no history of Axis 1 psychiatric disorders, as defined by the Structured Clinical Interview for DSM-IV, were recruited through the Volunteer Recruitment Pool at NKI as participants of other studies (Abeles and Gomez-Ramirez, 2014). All subjects provided informed consent and received a moderate fee for their time. This study was approved by the NKI Institutional Review Board.

### Stimuli (Human Experiments)

Gray scale images (132, 11 different individuals, four different emotions, three contrast levels) were used. Images were cropped in an oval window. Contrast was adjusted to 2, 8 and 57% root-mean-square contrast within the window. All images were presented centrally on a Phillips CRT monitor located 114 cm in front of participants. Major and minor axes of stimuli subtended 5 × 7 degrees of visual angle. A flower stimulus, enclosed in the same oval window as the faces, served as the target stimulus of the behavioral task. Only responses to faces of the highest contrast were analyzed for the present article.

### Behavioral Procedures (Human Experiments)

Face images were presented in random order, with infrequent (10% of times) target stimulus (Figure 1B). All images were presented for 500 ms. Inter-stimulus intervals were uniformly jittered between 900 and 1,100 ms. Participants were required to press a button for the flower image. A block of 120 trials lasted for approximately 3 min. All participants completed 30 blocks.

### Electrophysiological Recordings (Human Experiments)

Electroencephalography was acquired from 64 scalp electrodes using an ActiveTwo system (BioSemi, Amsterdam, The Netherlands). Data were digitized at 512 Hz, recorded relative to a common average reference during acquisition, and re-referenced offline to the average of all electrodes.

### Data Analysis (Human Experiments)

The data were filtered at 110 Hz, down-sampled at 256 Hz, separated into epochs (−100 to 500 ms), baseline corrected from −100 ms to stimulus onset, and an artifact rejection criterion of ±120 μV was applied to data from all sites. Epochs of responses to high contrast stimuli from all blocks were averaged. Signal at Fz was re-referenced to TP8. This was done to calculate ERP between positions that flank generators of N1, similar to the paired positions of electrodes in monkey experiments. ERP from each subject was highpass filtered at 0.5 Hz and averaged across subjects (*n* = 21). Confidence intervals were derived by boostrap (1,000 resamples). Visualization of scalp topography and estimation of N170 source was done using Brainstorm (Tadel et al., 2011). For source estimation, we used default Bio Semi 64 electrode positions and ICBM152 template of brain anatomy, and applied wavelet-based maximum entropy on the mean (wMEM) algorithm to the grand mean ERP.

## Results

### ERP to Faces in Monkeys

Data were obtained from two monkeys trained to attend to series of repeating AV vocalizations (standards) and to detect “oddballs” that differed from the standards in face, voice or both. During sessions of scalp recordings, monkeys made false alarms on standard (nontarget) stimulus presentation trials at low rates (Monkey G: 0.6, 0.25, and 0.51%, Monkey W: 0.39, 0.35, and 0.42% for Tasks 1, 2 and 3, respectively), and responded to targets approaching 100% accuracy.

As several comparative imaging studies have suggested the correspondence between the monkey STS region and the human FFA (Tsao et al., 2008; Pinsk et al., 2009), we chose electrode positions for monkey scalp recordings to maximize detection of signals generated by cortical areas in the banks of the STS. Cortical population neuronal activity can be addressed in scalp signals, depending on the configuration of the structure and the magnitude of activity (e.g., Ng et al., 2013). As STS runs through the temporal lobes on a track that is elevated several millimeters from the horizontal plain (Kajikawa et al., 2015), and cortical columns are oriented orthogonal to the cortical plane, it is expected that ERP components generated by STS cortices would have positive and negative polarities above and below the STS. We placed scalp electrodes approximately at FCz and FT9 or FT10, the latter two located just anterior to the left and right ears respectively (Figure 1A inset). Face stimuli activate wide cortical regions not limited to STS (e.g., occipital visual areas and inferior temporal gyrus). While those activities are volume conducted to any scalp positions to different degrees, the influences of those cortical areas on the scalp potential between the electrode positions are presumably minor due to distance and cortical orientation unless the STS response to face is weak. Also, the electrode positions avoided placement of electrodes on skin directly above the temporalis muscle, thus reducing electromyographic artifacts.

Figure 2A shows visual evoked potential responses to faces (face-ERP) during *Task 1*. While several faces were presented in every session, responses to different faces were averaged together since scalp ERP responses, like intracranial local field potential (LFP) responses (Hoffman et al., 2008), do not differ between faces. The face-ERP began with an initial positive component (mP1), followed by a large negative peak (mN1) and a secondary positive peak (mP2) during both tasks. While these responses to faces have not been assessed previously using scalp recordings in monkeys, the morphology, timing and sequence of the N1 and P2 components were similar timing to those of the N100 and P180 components of ERPs to faces recorded in tissues above STS in the temporal lobe (Anderson et al., 2008; Hoffman et al., 2008; Matsuo et al., 2011; Turesson et al., 2012; Kajikawa et al., 2017), although the smaller P1 components are detectable only near the STS (Kajikawa et al., 2017). The spatiotemporal patterns of both scalp and intracortical field potentials are consistent with generators underlying N1 and P2 being located in the lower bank of STS. The same observations suggest that P1 generators are located in areas outside of those examined here.

In *Task 1*, monkeys needed to monitor all abrupt visual events that were potential behavioral targets, like those in human experiments of the present study. However, in many monkey studies of face responses, animals were either anesthetized (Desimone et al., 1984; Tanaka et al., 1991; Tsao et al., 2008) or only required to maintain fixation during stimuli (Sugase et al., 1999; Tsao et al., 2003; Pinsk et al., 2005). In *Task 2*, which was designed to observe the effect of the face movement onset rather than face appearance, an abrupt face event occurred once in each trial under the more passive condition requiring only that the monkey maintain gaze on the stimulus (Figure 1A). Figure 2B shows face-ERPs during *Task 2*. Again, responses occurred with a sequence of mP1, mN1 and mP2. All 3 peaks appeared with similar timing regardless of the task conditions (Table 1). The additional negative peak between mN1 and mP2 during *Task 1* is presumably due to behavioral demand of the task to prepare manual response, or additional response to movie clips’ motion.

**Table 1:** Latencies of P1, N1 and P2.

		Left			Right		
Task	Monkey	P1 (ms)	N1 (ms)	P2 (ms)	P1 (ms)	N1 (ms)	P2 (ms)
1	G	62.5	114.5	205.5	64.0	115	206
	W	57.5	105.5	193.0	-	-	-
2	G	76.5	112.5	192.5	67.5	113.5	192.5
	W	50.5	113.5	173.0	52.5	109	180
∣rule
Humans	-	125.60 (15.2)	193.0 (13.0)	283.2 (29.6)	124.2 (17.2)	191.1 (12.2)	286.9 (25.0)

*Peak latencies of P1, N1 and P2 of face-ERPs measured at the scalp in different subjects and tasks*.

**Figure 3:**
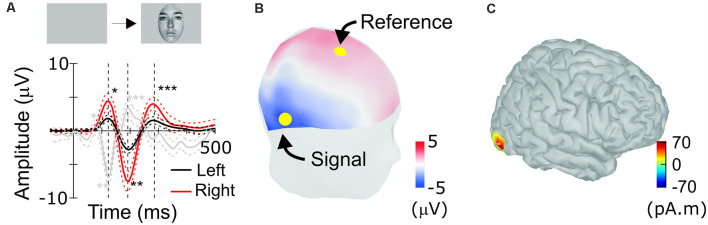
Scalp response to faces in humans. **(A)** Human face-evoked scalp potential response between channels Fz and TP8 (red), as indicated by yellow dots in **(B)**, and between channels Fz and TP7 (black; *n* = 21, averaged). Single, double and triple asterisks label peaks corresponding to the occipito-temporal hP1, hN1 (N170), and hP2, respectively. Dotted lines are 95% confidence intervals (bootstrap, 1,000 resamples). **(B)** Positions of scalp electrodes for recordings. The human scalp is colored to show scalp topography of potentials at the timing of N170. **(C)** Estimated location of the source of N170.

N170 and FFA activation in humans have biases on right side (Kanwisher et al., 1997, see also Figure 3). Lateralized biases have not been examined in monkeys. While Monkey W’s right side was not examined during *Task 1*, bilaterally recorded ERPs in Monkey G during *Task 1* had mN1 amplitudes of −0.26 (SD: 1.3) μV and −0.24 (SD: 1.1) μV, in right and left side respectively, that did not differ significantly (Figure 1A; *p* = 0.27). In contrast, the peak amplitude of mN1 during *Task 2* were −0.59 (SD: 3.1) μV and −0.47 (SD: 1.0) μV in right side of Monkeys G and W, and −0.36 (SD: 2.0) μV and −3.0 (SD: 1.3) μV in left side of them. mN1 in right side were significantly larger (Figure 1B, *p* = 0.012 and *p* = 0 for Monkeys G and W, respectively). While our mixed results are inconclusive requiring further studies, they suggest a possibility of a right-side bias in face responses of macaque monkeys like that seen in humans.

If mN1 and mP2 observed at scalp are generated in STS, these responses are expected to have preferences to complex visual objects like face and body parts like visual neurons in STS (Pinsk et al., 2005, 2009). In *Task 3*, we examined scalp responses to simple circles of a dimension similar to faces in *Tasks 1* and *2* (Figure 2C). While visual monitoring was irrelevant in *Task 3*, face responses in STS occur irrespective of visual attention (Tsao et al., 2003). Initial positive peak component appeared earlier than face responses, presumably due to the higher brightness of the circle than faces. However, in the temporal period immediately following negative and positive peak, components were sluggish. Absence of peaks similar to mN1 to faces in response to circles is consistent with previous findings that responses of STS neurons to simple shapes are weak compared to faces (Perrett et al., 1982; Desimone et al., 1984; Sugase et al., 1999). Additionally, separate intracranial recordings from same animals (Kajikawa et al., 2017), along with other studies (Hoffman et al., 2008; Turesson et al., 2012), showed visual field potentials to faces in the temporal lobe had peak components similar to mN1 and mP2 and their generators were identified in STS.

Since *Task 3* required only auditory attention to detect targets, less visual attention, in addition to object complexity, could have reduced the visual response. To address the effect of attention, face-ERPs in Monkey W’s left side were examined during *Task 2* under a condition in which all targets were auditory oddball targets, with no change in visual modality. Recordings were performed at early stages of trainings before introducing visual targets in the tasks. Even when visual attention was not yet required, face-ERP occurred with mN1 at 106 ms (gray lines in Figure 2B). These observations suggest that attentional considerations are insufficient to explain the absence of strong responses to simple circles in cortices including STS that were flanked by scalp electrode locations. Taken together with those findings, the results in the present study support the possibility that cortical regions in STS generated both mN1 and mP2 face responses on the scalp.

### ERP to Faces in Humans

For comparison, ERPs were also recorded from humans performing similar tasks to detect visual targets in sequentially presented faces (Abeles and Gomez-Ramirez, 2014). Typically, human ERPs are re-referenced to the grand average or the signal recorded at the mastoid (Kayser and Tenke, 2010). However, as we derived macaque ERP between positions that flank STS, we calculated humans’ face-ERP between electrodes that flanked the source of N170 (Figure 3C). Electrodes were selected at positions Fz and TP8, considering the source cortical position and orientation. Face-ERPs derived in humans have a sequence of peaks: hP1, hN1 followed by hP2, with hN1 being the largest (Figure 3A). The topography of mean human scalp ERP at the timing of N170, depicted in Figure 3B, shows typical scalp pattern of occipito-temporal negative (N170) and fronto-central positive (VPP) peaks (Rossion, 2014), even though peak latencies were slower than typical latencies in the N170 literature. The source derived from the N170 topography is located approximately at the position of the FFA (Figure 3C). ERPs to faces were larger on the right side, consistent with the right-side bias observed in most prior studies of face responses in humans.

Face-ERPs recorded from humans display a stereotyped temporal pattern of components that strongly resembled the succession of components recorded from the macaque. The largest face-ERP component in both species is the N1. Face-ERP components differ in their peak timing between species (Table 1); human ERP components have longer peak latencies than those of macaques (note the difference in the time scale between Figure 2 and Figure 3A). The ratio of peak latencies of the face-ERP were close to 3/5 for pairs of mN1-hN170 and mP2-hP2 components. These latency differences are in line with the general temporal scaling of sensory responses between these species (the 3/5 rule, see “Discussion” section).

## Discussion

### Factors Impacting Cross-species Comparisons

Comparisons of brain responses between species generally have multiple problems due to differences in behavioral conditions, stimuli, recording techniques and brain structures. Difference in every factor can affect the comparison.

In the present study, humans and monkeys performed similar tasks. Though the monkey tasks interleaved trials of different modalities, we used only the trials with visual-alone stimuli that, like the human task, required only visual attention. There were other minor methodological differences between the human and monkey studies (e.g., to indicate target detection, monkeys released a joystick while humans pressed a button), but these are not considered to be consequential. Further, while the context of face events in trials differed between Tasks 1 and 2 in monkey experiments, face-ERPs appeared similarly between tasks, and face activation in STS has previously been shown to be stable across behavioral conditions (Tsao et al., 2003; Kajikawa et al., 2017). The temporal patterns of human face-ERP currently observed were also similar to those in other studies using different tasks and behavioral conditions (Herbert et al., 2013; Syrjänen et al., 2018). Under different conditions, equivalent dipole sources of N170 are localized at FFA (Deffke et al., 2007; Soto et al., 2018; Gao et al., 2019). Thus, even though cortical activation by faces would be widespread, the source contributing most to N170 is likely FFA in humans and FFA activation presumably occurs across different cognitive conditions.

Our studies in both species used close-up views of conspecific faces as stimuli. Since they differed in color (gray and color in humans and macaques, respectively), cross species comparison could be confounded in activated cortical areas related to color-coding. However, color-coding areas and face coding areas are adjacent to one another in both macaque IT (Lafer-Sousa and Conway, 2013) and human occipito-temporal regions (Lafer-Sousa et al., 2016). Also, face responses in face areas are larger than additional face responses in color areas (Lafer-Sousa and Conway, 2013). Thus, regardless of additional activation in monkeys due to use of coloring in the face stimuli, the relative contributions of color-coding areas to scalp response is considered to be minor.

While recording techniques were made equivalent between species by using similarly low impedance electrodes on the scalp, we used different pairings of electrodes between species for deriving ERPs. These species differ in the locations and orientations of major cortical activations in response to faces, FFA in humans and IT, particularly area TEa, the rostral part of the lower bank of STS (Pinsk et al., 2005; Tsao et al., 2006; Kajikawa et al., 2017), in macaques. FFA is located at the occipito-temporal surface of brain in humans, and area TEa lies in the lower bank of the STS in macaques. In area TEa, which is a long band elongated posterior-anteriorly, response latency increases from posterior to more anterior recording sites (Freiwald and Tsao, 2010).

However, during widespread activation, temporal components of the ERP may not correspond to the peak timing of underlying individual components but appear as the sum of those components due to volume conduction (Luck, 2014). Waveforms due to volume conduction are dominated by those of momentarily strong activations (Kajikawa et al., 2017). The extant literature on face responses supports the idea that TEa and occipitotemporal cortex are the epicenters of activity in macaques (Tsao et al., 2003, 2006) and humans (Halgren et al., 1999; Tsao et al., 2003; Grill-Spector et al., 2004), respectively. Thus, temporal components in macaque face-ERPs mostly reflect activations in those cortical regions.

There are many studies of face responses in both humans and macaques. Human studies show consistent bias that cortical face response is larger on the right side (Rossion et al., 2003; Hildesheim et al., 2020). Such bias has not been addressed in macaques. Our results point out the possibility of the presence of similar right bias in macaques’ face responses as well. However, it did not appear consistently between task conditions, and the task conditions were not same as those of human studies. Face response characteristics like amplitude and latency are presumably sensitive to various parameters of cognitive and stimulus conditions. Also, the extent of those properties shared between humans and macaques is still unknown. Further studies are needed to fully characterize similarities and relationships of visual responses between species.

### Latency Offset Between Macaque and Human ERP Components

Peak latencies of mN1 and mP2 were 105–115 ms and 180–205 ms, respectively. Those were close to corresponding peak latencies of intracortical visual filed potentials recorded above STS in macaques (i.e., 100 ms in Hoffman et al., 2008; and 120 ms in Kajikawa et al., 2017). However, hN1 latency, 190~195 ms, was longer than typical values around 170 ms for unknown reasons. By allowing the latencies of those peaks to be labile ranging from 100 to 120 ms for mN1 and from 170 to 195 for hN1, ratio of mN1 and hN1 peak latencies ranges 0.5–0.7. Thus, ratio of peak latencies fluctuates around 0.6. This ratio of peak timing is similar to other sensory responses generated in primary sensory areas as those have peak latency ratio of approximately 3/5 between macaques and humans across sensory modalities (Schroeder et al., 1995, 2004). For example, the peak latency of the occipital P1 component elicited by pattern reversal stimuli is about 60 ms in macaques (Schroeder et al., 1991) and 100 ms in humans (DeVoe et al., 1968). Peak latency of fronto-central auditory P1 is ~30 ms in monkeys (Steinschneider et al., 1992) vs. ~50 ms in humans (Liégeois-Chauvel et al., 1994; Howard et al., 2000). Somatosensory evoked potentials to electrical stimulation to median nerve stimulation have P1 at 12 ms in macaques (Arezzo et al., 1981; Peterson et al., 1995), and 20 ms in humans (Allison et al., 1989).

Interestingly, chimpanzees have discretely distributed face-selective areas including STS and fusiform gyrus like humans (Parr et al., 2009), and they also generate a face-ERP at FCz with a negative peak at 140 ms, similar to the macaque N1 (Fukushima et al., 2010). Thus, in both anatomical and physiological terms, chimpanzees appear to be an intermediary species between monkeys and humans. Functional divergence of parietal/temporal regions may account for displacement of fusiform gyrus in apes from IT in monkeys (Orban et al., 2004).

Recent intracranial recordings in humans showed visual responses in regions that are presumably upstream and downstream of FFA in the pathway of visual signal transduction. Self et al. (2016) estimated onset latency of MUA responses in V2/3 was about 60 ms and comparable to or slightly shorter than SUA responses latencies 70–80 ms of equivalent visual areas in macaques (Schmolesky et al., 1989). Those results suggest that the latent period of visual responses up to occipital visual areas may not differ between macaques and humans. However, unit firing onset is always earlier than peak latency of slower electrical signals like field potentials and those signals may originate in different cortical layers (Leszczyński et al., 2020). ECoG signals near those areas have peak latencies 100~120 ms similar to visual P1 on the occipital scalp (Yoshor et al., 2007). It is notable that same study showed ECoG in the zone near FFA had peak latency 152 ms to small simple stimuli. The latency is in the range of N170 latency to face stimuli. Thus, the peak latency of occipitotemporal response may not vary regardless of stimulus type.

It has been shown that amygdala connects to STS anatomically in macaques (Stefanacci and Amaral, 2000, 2002), and functionally in humans (Pitcher et al., 2017). Minxha et al. (2017) compared single unit responses of amygdala in both monkeys and humans, and found onset latencies of 100 and 177 ms, and peak latencies 209 and 322 ms. Ratios of both onset and peak latencies were comparable to 3/5. Thus, difference of visual response latency between species may extend beyond STS.

Lastly, the peak latency of N170, labeled as hN1 in the present study, is not exactly 170 ms but variable across human studies. The variability is presumably attributable to different experimental conditions. Similarly, stimulus conditions were also not identical between monkey and human experiments in the present study, e.g., the gray scale image used in human experiments had presumably had lower contrast than those in monkey experiments, which resulted in an average hN1 latency longer than 170 ms. Comparisons across experimental conditions and between species need to factor in these differences. Thus, the ratio of monkey to human latency, argued here as 3/5, may not be exact but is likely somewhat variable, while its value may fall in a range around 3/5.

## Conclusion

Scalp face responses in monkeys and humans have different topographic patterns attributable to differences in the positions of face-activated cortical areas. Regardless, the clear correspondence between macaque and human ERP components supports the use of the macaque as a model of delineating the neuronal substrates of face ERPs recorded from the scalp in humans (Kajikawa et al., 2017). Moreover, the peak latencies of corresponding monkey and human face ERP components scale at around 3/5 ratio between these species generally across studies, similarly to other sensory responses, suggesting that the relative sensory processing speed are preserved across modalities, as well as higher order stages of the visual pathways.

## Data Availability Statement

The raw data supporting the conclusions of this article will be made available by the authors, without undue reservation.

## Ethics Statement

The studies involving human participants were reviewed and approved by Institutional Review Board of the Nathan Kline Institute. The patients/participants provided their written informed consent to participate in this study. The animal study was reviewed and approved by Institutional Animal Care and Use Committee of the Nathan Kline Institute.

## Author Contributions

YK conceived the idea and performed the monkey experiments. IA, PB, and MG-R performed the human experiments. YK and JO analyzed the data. YK, PB and CS wrote the manuscript. All authors contributed to the article and approved the submitted version.

## Conflict of Interest

The authors declare that the research was conducted in the absence of any commercial or financial relationships that could be construed as a potential conflict of interest.

## Abbreviations

ERP: event related potential
FFA: fusiform face area
OFA: occipital face area
STS: superior temporal sulcus
VPP: vertex positive potentials.
